# Chronic idiopathic axonal polyneuropathy: Prevalence of pain and impact on quality of life

**DOI:** 10.1002/brb3.1171

**Published:** 2018-11-25

**Authors:** Panagiotis Zis, Ptolemaios G. Sarrigiannis, Dasappaiah G. Rao, Channa Hewamadduma, Marios Hadjivassiliou

**Affiliations:** ^1^ Academic Department of Neurosciences Sheffield Teaching Hospitals NHS Foundation Trust Sheffield UK

**Keywords:** chronic idiopathic axonal polyneuropathy, CIAP, pain, quality of life

## Abstract

**Background and Aim:**

Chronic idiopathic axonal polyneuropathy (CIAP) is a term describing axonal neuropathies of insidious onset, with slow or no progression of the disease over at least 6 months and with no etiology being identified despite appropriate investigations. We aimed to establish the prevalence of pain in patients with CIAP and investigate the impact of pain on quality of life (QoL).

**Methods:**

All consecutive patients with CIAP attending a specialist neuropathy clinic were invited to participate. Pain was assessed via the DN4 questionnaire and the visual analogue scale (VAS). Overall Neuropathy Limitations Scale (ONLS) was used to assess the severity of neuropathy. The SF‐36 questionnaire was used to measure participants’ quality of life.

**Results:**

Fifty‐five patients with CIAP were recruited (63.6% male, mean age 73.4 ± 8.7 years). Based on the DN4 questionnaire, peripheral neuropathic pain was present in 33 patients (60.0%). After having adjusted for age, gender and disease severity pain showed significant negative correlations with the energy/fatigue domain of QoL (*β* = −0.259, *p* = 0.049), with the emotional well‐being domain (*β* = −0.368, *p* = 0.007) and the general health perception domain (*β* = −0.356, *p* = 0.007).

**Conclusion:**

Pain is very prevalent in CIAP and is associated with poorer emotional well‐being, worse general health perception, and increased fatigue.

## 1 INTRODUCTION

Chronic idiopathic axonal polyneuropathy (CIAP) is a term describing neuropathies where neurophysiology reveals axonal damage, their onset is insidious and shows slow or no progression of the disease over at least 6 months and no etiology is identified despite appropriate investigations (Zis, Sarrigiannis, Rao, Hewamadduma, & Hadjivassiliou, 2016).

Sensory symptoms are more prominent in CIAP and occur earlier in the course of the disease (Teunissen et al., 1997). Numbness is the most commonly reported presenting symptom (Teunissen et al., 1997; Wolfe et al., 1999); however, pain can also be very prevalent in CIAP (Zis et al., 2016). Robust epidemiological data are lacking, and the reason for this is twofold. Firstly, in the majority of the available studies on CIAP, the diagnostic investigations are incomplete. Secondly, when reported, pain prevalence and characteristics were not the primary aim of such studies.

Pain can increase the global burden of the disease and might affect the patients’ quality of life (QoL; Rice, Smith, & Blyth, 2016; Teunissen, Eurelings, Notermans, Hop, & Gijn, 2000). Overall, patients with CIAP have worse QoL compared to the general population (Lindh, Tondel, Persson, & Vrethem, 2011); however, it is unclear if pain has an additional impact on the QoL in patients with CIAP.

The purpose of our cross‐sectional study was to establish the prevalence of peripheral neuropathic pain in patients with CIAP and investigate the effect of pain on QoL.

## 2 METHODS

### 2.1 Procedure and participants

This was a cross‐sectional study. Patients were recruited from a tertiary specialist neuropathy clinic.

To be enrolled, the patients had to meet the following inclusion criteria: (a) clinical diagnosis of peripheral neuropathy (PN), confirmed on nerve conduction studies (NCS), (b) absence of other risk factors for developing PN (i.e., diabetes, vitamin deficiencies, exposure to neurotoxic agents), (c) normal results on an extensive diagnostic work‐up detailed below, (d) able to provide a written informed consent, and (e) not having a family history of PN.

The study protocol was approved by the local ethics committee.

### 2.2 Measures

Demographic characteristics included age and gender.

All patients went through extensive investigations for possible causes of PN (Zis et al., 2016). The tests included full blood count, erythrocyte sedimentation rate, kidney function tests (i.e., urea and electrolytes), liver function tests, thyroid function tests, immunoglobulin levels and electrophoresis, serum angiotensin converting enzyme, HbA1c, B12, and folate. We performed extensive immunological tests that included ANA, gliadin IgA and IgG, endomysial, transglutaminase, anti‐dsDNA and ANCA antibodies, rheumatoid factor, and ENA panel. We also tested patients for any evidence of HCV and HIV infections. Finally, patients with rapidly progressing neuropathy were tested for any evidence of a paraneoplastic syndrome, including antineuronal antibodies and imaging studies (i.e., PET scan).

### 2.3 Neurophysiological assessment

All patients had detailed nerve conduction studies (NCS) that were performed by the same clinician on the day of the recruitment.

The following parameters were measured using Natus EMG equipment (Viking EDX): antidromic superficial radial sensory nerve action potential (SNAP), antidromic sural SNAP, antidromic superficial peroneal SNAP, orthodromic median SNAP, orthodromic ulnar SNAP, median compound muscle action potential (CMAP), ulnar CMAP, tibial CMAP, and common peroneal CMAP bilaterally. For all studies, temperature was above 32°C for the upper extremity and above 31°C for the lower extremity. The temperature was measured before and after each study on the dorsum of the hand and the foot. Frequency filters were 2 Hz (low) and 2 kHz (high) for the sensory studies and 3 Hz (low) to 10 kHz (high) for the motor studies. SNAPs were measured as peak‐to‐peak, whereas CMAPs were measured as onset‐to‐peak.

Based on the published normal values (Esper et al., 2005; Leis & Schenk, 2013) that we use in our department, all patients had evidence of axonal loss (attenuated sensory nerve action potentials and/or compound muscle action potentials) in at least two nerves tested (Johnsen & Fuglsang‐Frederiksen, 2000). No patients had evidence of demyelination, based on the following published criteria (Bergh & Piéret, 2004): (a) >150% prolongation of motor distal latency above the upper limit of normal values; (b) <70% slowing of motor conduction velocity below the lower limit of normal values; (c) >125% (>150% if the distal negative‐peak CMAP amplitude was <80% of the lower limit of normal values) prolongation of *F*‐wave latency above the upper limit of normal values; or (d) abnormal temporal dispersion (>30% negative‐peak CMAP duration increase) in two or more nerves.

The type of axonal neuropathy for all patients was determined based on the NCS and electromyography (EMG). The electrophysiological diagnosis of symmetrical sensorimotor axonal polyneuropathy was based on finding reduced distal sensory and/or motor amplitudes on nerve conduction studies and a distal to proximal gradient of re‐innervation or denervation on EMG (Albers, 1993). Sensory neuronopathy also known as sensory ganglionopathy (SG) a type of pure sensory neuropathy affecting the cell bodies of the sensory neurones located in the dorsal root ganglia was diagnosed based on the published neurophysiological criteria (Zis, Hadjivassiliou, Sarrigiannis, Barker, & Rao, 2017). In SG, complete absence or asymmetry in the sensory nerve action potentials predominates (Zis, Hadjivassiliou, et al., 2017).

The severity of neuropathy was assessed by the overall limitations neuropathy scale (ONLS), which is a validated scale that measures limitations in the everyday activities of the upper and lower limbs (Graham & Hughes, 2006).

### 2.4 Pain evaluation

Neuropathic pain was assessed via the DN4 questionnaire (Bouhassira et al., 2005). DN4 is a clinician administered screening tool consisting of 10 yes/no items. A score of equal to or greater than 4 is considered as diagnostic of neuropathic pain.

Pain intensity was assessed via a Visual Analogue Scale (VAS) ranging from 0 (no pain at all) to 10 (worst pain you can imagine). Pain intensity was assessed for two time points: pain at recruitment and maximum peripheral pain experienced during the disease course. Only patients reporting pain intensity of equal to or greater than 4 at any point were considered to suffer from a painful neuropathy.

The 36‐Item Short Form Survey (SF‐36), a self‐reported measure of health status and quality of life (Sykioti et al., 2015), was used to determine patient health‐related quality of life. SF‐36 covers nine health and QoL domains. These domains include physical functioning; role limitations due to physical health; role limitations due to emotional problems; energy/fatigue; emotional well‐being; social functioning; pain; general health; and health change. Each item is measured using a Likert‐type scale. Scores were converted and analyzed according to the marking guidelines for the SF‐36, such that higher scores (out of a total of 100 for each domain) constitute better health‐related quality of life in this domain.

### 2.5 Statistical analyses

A database was developed using the Statistical Package for Social Science (version 23.0 for Mac; SPSS). Frequencies and descriptive statistics were examined for each variable. Comparisons between patients with painful CIAP and patients with painless CIAP were made using Student's *t* tests for normally distributed continuous data, Mann–Whitney's *U* test for non‐normally distributed, and chi‐square test or Fisher's exact test for categorical data.

Where differences with a *p* value of lower than 0.10 were found, these variables were entered into linear regression models, with the QoL domain score being the dependent variable. All accuracy and generalization assumptions for the model were checked.

The level of statistical significance was set at the 0.05 level.

## 3 RESULTS

### 3.1 Study population

Fifty‐five patients with CIAP were recruited (63.6% male, mean age 73.4 ± 8.7 years). Forty‐seven patients (85.5%) had a symmetrical length‐dependent sensorimotor axonal PN, and eight (14.5%) had a sensory ganglionopathy. Mean age at neuropathic symptoms’ onset was 63.1 ± 110.5 years (ranging from 25 to 85 years). Overall ONLS scores ranged from 1 to 7 (mean 3.3 ± 1.6).

Eleven patients (20%) reported pain to be the first symptoms of the PN (six reported a sharp pain, two reported allodynia, and three reported a burning painful sensation).

Based on the DN4 questionnaire, and a VAS score of at least 4/10, neuropathic pain at any point was present in 33 patients (60.0%). Based on a VAS score of at least 4/10, pain was present on examination in 20 patients (36.4%), whereas the other 13 patients reported spontaneous neuropathic pain, occurring randomly during the day. The pain intensity on examination ranged from 0 to 10 (mean 4.1 ± 2.9), when the maximum pain experienced ranged from 5 to 10 (mean 8.6 ± 1.6).

None of the 22 patients reporting no pain were on anti‐neuralgic treatment.

### 3.2 Univariate analysis

Table 1 summarizes the demographic and the clinical characteristics of the patients with painful and the patients with painless CIAP. The two groups did not differ significantly regarding age, gender, type of neuropathy, or disease duration. Patients with painful neuropathy had a more severe neuropathy compared to patients without pain (mean ONLS overall score 3.7 ± 1.5 vs. 2.8 ± 1.5, *p* = 0.024).

**Table 1:** Characteristics of our study population

	Painful CIAP (*n* = 33)	Painless CIAP (*n* = 22)	*p* value
Demographics			
Age, in years (*SD*)	73.5 (6.7)	73.3 (11.2)	0.910
Male gender (%)	19 (57.6)	14 (63.6)	0.252
Clinical characteristics			
Disease duration, in years (*SD*)	9.7 (7.5)	11.3 (7.1)	0.440
Type of neuropathy			0.876
Symmetrical length‐dependent PN (%)	28 (84.8)	19 (86.4)	
Sensory ganglionopathy (%)	5 (15.2)	3 (13.6)	
Neuropathy severity			
ONLS Arm score (*SD*)	1.5 (0.8)	0.9 (0.9)	0.015
ONLS Leg score (*SD*)	2.3 (1.0)	1.9 (1.2)	0.221
Total ONLS score (*SD*)	3.7 (1.5)	2.8 (1.5)	0.024
Quality of life modalities			
Physical functioning	36.4 (21.7)	58.6 (29.2)	0.002
Role limitations due to physical health	34.3 (23.8)	55.1 (33.6)	0.009
Role limitations due to emotional problems	66.9 (31.6)	80.7 (31.4)	0.118
Energy/Fatigue	35.6 (20.2)	50.9 (18.7)	0.007
Emotional well‐being	59.7 (23.2)	78.4 (14.0)	0.001
Social functioning	56.1 (32.0)	72.7 (29.3)	0.056
Pain	37.4 (21.5)	72.2 (24.4)	<0.001
General health	41.1 (22.7)	58.9 (15.4)	0.002
Health change	32.6 (18.2)	43.2 (11.4)	0.019

As shown in Table 1, apart from the domain of pain, patients with painful CIAP presented with significantly worse scores compared to patients with painless CIAP in the following quality of life modalities: physical functioning (*p* = 0.002), role limitations due to physical health (*p* = 0.009), energy/fatigue (*p* = 0.007), emotional well‐being (*p* = 0.001), general health (*p* = 0.002), and health change (*p* = 0.019), whereas there was a trend of statistically significant difference in the domain of social functioning (*p* = 0.056).

### 3.3 Multivariate analysis

Table 2 summarizes the multivariate linear regression models for all QoL domains, which showed statistically significant differences or a trend for a statistical significant difference. After having adjusted for age, gender and disease severity (total ONLS sore) pain showed significant negative correlations with energy/fatigue domain (*β* = −0.259, *p* = 0.049), emotional well‐being (*β* = −0.368, *p* = 0.007), and general health perception (*β* = −0.356, *p* = 0.007).

**Table 2:** Multiple linear regression models: effect of the pain on QOL measures in CIAP

		Physical functioning	Role limitations Due to physical health	Energy/Fatigue	Emotional well‐being	Social functioning	General health	Health change
Age	*β*	−0.120	−0.108	0.303	0.169	0.034	0.371	0.101
	*p*	0.240	0.342	0.018	0.189	0.776	0.003	0.436
Gender	*β*	0.162	−0.037	0.195	0.197	−0.008	−0.010	0.176
	*p*	0.111	0.743	0.118	0.122	0.944	0.931	0.173
Total ONLS score	*β*	−0.567	−0.519	−0.254	−0.084	−0.531	−0.180	−0.320
	*p*	<0.001	<0.001	0.055	0.528	<0.001	0.161	0.021
Pain	*β*	−0.208	−0.193	−0.259	−0.368	−0.100	−0.356	−0.194
	*p*	0.053	0.104	0.049	0.007	0.426	0.007	0.153

## 4 DISCUSSION

This cross‐sectional study demonstrates that three out of five patients with CIAP suffer from peripheral neuropathic pain secondary to their neuropathy. Very importantly, presence of pain is correlated with worse quality of life in the energy/fatigue domain, the emotional well‐being domain, and the general health perception domain.

Contrary to the common misperception that painful neuropathies are related to diabetes, our study strengthens the evidence that neuropathies of etiologies other than diabetes can be equally painful. The reported prevalence of pain in patients with diabetic PN, confirmed with NCS, is estimated to be 40%–50% (Ware, 1992). Previous systematic reviews have established that the prevalence of pain in paraneoplastic neuropathies is 58% (Didangelos, Doupis, & Veves, 2014) and in platin‐induced neuropathies is 46%, when referring to patients with established PN following chemotherapy completion (Zis, Paladini, et al., 2017). These figures are very similar to our findings, as in our cohort of CIAP patients, the estimated prevalence of peripheral neuropathic pain was 60%.

In 2010, Erdmann et al. described a case series of ninety‐one patients with CIAP and they reported that the overall prevalence of pain in CIAP is 69% (Brozou, Vadalouca, & Zis, 2017). However, when looking more specifically into the peripheral neuropathic pain, the reported prevalence dropped to 42% (Brozou et al., 2017). There are few possible explanations for the difference between the latter figure and the prevalence in our cohort.

Firstly, the mean age of our CIAP population was approximately 7 years higher compared to Edmann's cohort and pain has been shown to increase with age (Erdmann et al., 2010). Secondly, contrary to our study, Erdmann et al. provided no information about the clinical severity of the PN and therefore the reported prevalence may also vary because of the different PN severity of the two cohorts. In our study population, patients with pain had a more severe PN, as this was determined by the ONLS. Finally, perception of pain varies among individuals and depends on many variables, including genetic predisposition and gender (Fayaz, Croft, Langford, Donaldson, & Jones, 2016; McGrath, 1994).

A novelty of our study is that our cohort included patients with idiopathic sensory ganglionopathy, the second commonest type of axonal neuropathy. In total, one out of seven CIAP patients (14.5%) had a sensory ganglionopathy, rather than a symmetrical length‐dependent neuropathy, whereas in Edmann's cohort all patients had a symmetrical sensorimotor neuropathy. The univariate analysis showed that presence of pain does not depend on the neurophysiological type of CIAP.

In this study, we also described the QoL of patients with CIAP. In the univariate analysis, we found that patients with pain have significantly worse scores on all QoL domains apart from the domain of role limitations due to emotional problems (not statistically significant) and the domain of social functioning (trend for statistical significance). This observation differs to what has been reported by Erdmann et al. who found only significant correlations between pain and the physical functioning domain (Brozou et al., 2017). Again, the demographic and clinical differences between the two cohorts may account for this difference.

Another novelty of our study is that we performed multivariate analyses and were able to identify correlations between pain and the QoL domains after having adjusting for age, gender, and PN severity. The domains where pain plays independently a significant role are the energy/fatigue, the emotional well‐being, and the general health perception domains. This is particularly important as it highlights that pain plays a significant role in the emotional state of the patient and might cause or enhance feelings of anxiety and depression, regardless of age, gender, and disease severity (Zis, Daskalaki, et al., 2017). Therefore, questioning about the presence of pain in CIAP patients is crucial as by symptomatic treatment of pain the QoL of patients might improve. A future prospective study comparing patients on symptomatic treatment with those not on any treatment may be able to confirm this.

Treatment of PN depends on the etiology and primarily aims to stop the disease progression. As CIAP is by definition of unknown etiology, management of CIAP targets to symptomatically relieve the symptoms that patients’ experience. With regards to neuropathic pain, no completed studies for the evaluation of the effectiveness of various anti‐neuralgics in the treatment of CIAP exist. Patient Assisted Intervention for Neuropathy: Comparison of Treatment in Real Life Situations (PAIN‐CONTRoLS) is a PCORI initiative, through the University of Kansas, aiming to address this particular question; the results of this study (registration NCT02260388) are expected to be available in late 2018. Therefore, until then, the management of pain in CIAP should be based on the published guidelines for the management of neuropathic pain (Vadalouca et al., 2012).

Our results should be interpreted with some caution, however, given the limitations of our design. This is a cross‐sectional observational study comparing CIAP patients with and without peripheral neuropathic pain based on patients attending a specialized clinic, and the results may not be generalizable to other settings. For example, in our study, we used the ONLS to determine the clinical severity of the neuropathy, whereas other departments use other scales. The independent role of pain in the overall QoL might differ if other tools are used, something that could be investigated in a future study. Furthermore, our cohort included patients with large fiber axonal peripheral neuropathies only. Idiopathic small fiber neuropathy is another area that merits further consideration, as it is a particularly painful condition. Finally, we were able to demonstrate that the presence of pain is an independent determinant of QoL after having adjusted for age, gender, and neuropathy clinical severity as this was assessed via the ONLS. The latter evaluates fine movements of the upper limbs and gait. Thus, primarily, it depends on the motor involvement secondary to neuropathy or can be abnormal in cases of severe sensory ataxia leading to gait impairment. Therefore, we have not adjusted for the presence of other sensory symptoms such as itchiness and tingling, though in our clinical practice patients rarely complain about how other sensory symptoms than pain affect their lives.

In conclusion, pain is very prevalent in CIAP and significantly impacts on QoL. Optimum management of pain in such patients might prove beneficial in improving QoL. Future prospective studies are needed to explore further the relationship between pain and QoL.

## CONFLICT OF INTEREST

None.
